# Evaluation of Electrocardiogram-Gated Computed Tomography Angiography to Quantify Changes in Geometry and Dynamic Behavior during the Cardiac Cycle of the Nellix Endovascular Sealing System

**DOI:** 10.1155/2023/3721185

**Published:** 2023-06-21

**Authors:** Leo H. Van Den Ham, Talar Lazarian, Erik Groot Jebbink, Jan-Willem Lardenoije, Clark J. Zeebregts, Michel M. P. J. Reijnen

**Affiliations:** ^1^Department of Surgery, Rijnstate, Arnhem, Netherlands; ^2^Multi-Modality Medical Imaging Group, TechMed Centre, University of Twente, Enschede, Netherlands; ^3^Department of Surgery, Division of Vascular Surgery, University Medical Center Groningen, University of Groningen, Groningen, Netherlands

## Abstract

**Background:**

The Nellix endovascular sealing system (EVAS) was a unique concept with regard to its sealing concept that failed, related to high migration rates. We investigated the changes in aortoiliac morphology during the cardiac cycle before and after EVAS using electrocardiography (ECG)-gated CT.

**Methods:**

Eight patients scheduled for EVAS were prospectively enrolled. ECG-gated CT scans were made pre- and postoperatively. Measurements were performed in the mid-systolic and mid-diastolic phases. Endpoints were changes in infrarenal aortoiliac morphology postoperatively compared to preoperatively and their changes in the cardiac cycle.

**Results:**

Both pre- and postoperatively, there were no changes during the cardiac cycle. EVAS caused an increase in neck diameter and surface in both phases (*p* < 0.001). EVAS increased the luminal AAA volume (*p* < 0.001), with a decrease in thrombus volume (*p* < 0.001) in both phases and an increase in total volume (*p* < 0.001) in the systolic phase. During follow-up, one patient presented with >5 mm migration. There were no differences in the movements of this patient compared to the remaining patients.

**Conclusion:**

The cardiac cycle had a very limited effect on the aortoiliac dynamics before and after EVAS and, therefore, there is probably not a role for ECG-gated CT in enhanced surveillance programs. EVAS itself has a significant impact on anatomy, particularly the neck diameter, length, and volumes of the AAA.

## 1. Introduction

After its commercial introduction in 2013, the Nellix endovascular sealing system (EVAS) was rapidly adopted in the vascular community, but late complications such as proximal endoleaks and proximal migration were observed. This first led to a refined IFU which significantly reduced the applicability of the technique [1] and later the final withdrawal from the market in May 2022. The system was distinct from other systems by sealing the aneurysm with polymer-filled endobags and thus fixating inside the aneurysm sac itself. Lateral bending due to incomplete endobag filling in AAAs with large thrombus volumes was suggested as a driving force behind distal migration. The polymer-filled endobags increased the rigidity of the stents and could thus reduce the risk on migration [1, 2].

Excessive motion of the aorta, in both the longitudinal and lateral directions, has been suggested as a risk factor for endograft migration after conventional EVAR [3]. This could also be an important factor after EVAS, where downward forces on the polymer shell and lateral forces through curves in the stent frames may play a role. The current gold standard for both preoperative sizing and follow-up after EVAR/EVAS is static CT imaging. This method relies on static imaging of a dynamic process; hence, the accuracy of these measurements is uncertain.

A previous study has examined the translation and aortic curvature of EVAS during the cardiac cycle, but no quantitative anatomical parameters have been examined previously [4]. This study aimed to get more insight into the effect of EVAS on the motion of the aortoiliac tract by measuring changes in ECG-gated computed tomography (ECG-gated CT) before and after EVAS. This is still clinically relevant, as many patients that were treated in the past are still in enhanced surveillance programs [5].

## 2. Methods

### 2.1. Study Design

This was a single-center prospective observational study. Eight patients who were scheduled for EVAS were prospectively enrolled in this study. Each patient underwent an ECG-gated CT preoperatively and six weeks after EVAS. The preoperative CT was used for procedural planning. Patient demographics as well as procedural data were collected and all pre-and postoperative imaging was studied. The study was conducted according to the principles of the Declaration of Helsinki (64^th^ WMA General Assembly, Fortaleza, Brazil, 2013) and in accordance with the applicable guidelines, regulations, and acts. For this prospective study, approval by an independent institutional review board was obtained, and each patient had given written informed consent before inclusion in this study. Patient scan information and data were stored anonymously. The study was registered on clinicaltrials.gov with ID: NCT02438605.

### 2.2. Imaging

An (electrocardiography) ECG-gated CT provides images at various stages of the cardiac cycle. By analyzing these images, it is possible to measure the movements of the stent graft and the arteries during the diastole and systole cycles of the heart. A Phillips Brilliance iCT 256-slice scanner was used to perform the ECG-gated CT scans in all cases, with dose modulation (automatic), using a standardized protocol, and during a single breath hold. Radiation exposure was tube voltage 120 kV, reference mAs 400 mAs, and pitch 0.34. The scan was made at 10 time points during the cardiac cycle and at the highest atrial pressure (20%) and lowest atrial pressure (78%), which were the chosen phases for further assessment. The studied slice thickness was 0.9 mm, and Xenetix 300 (Guerbert GmbH, Sulzbach, Germany) was used as iodine contrast medium and adapted according to the patient's weight. The scan was started using bolus triggering at the height of the aortic arch at a threshold of 150 HU over baseline.

### 2.3. Analysis

All images were analyzed using 3Mensio (Pie Medical Imaging, Maastricht, The Netherlands). Measurements were performed based on anatomical landmarks, after creating a central lumen line (CLL) with the workflow assistant in 3Mensio. The measurement protocol was partially based on reporting standards and previous studies regarding CTA-imaging after EVAR [6]. In order to create the CLL, a 3D reconstruction and semiautomatic segmentation of the abdominal aorta, renal, and iliac arteries were needed in two phases of the cardiac cycle, 20% and 78% phases, per patient. The CLL was required to be able to view the images perpendicular to the vessel/arterial axis. The CLL was created semiautomatically, but manually checked and confirmed, by sliding through every plane on snake view and correcting where necessary.

Measurements on morphology were divided into three areas, including the infrarenal neck, the AAA sac, and the common iliac arteries. The start of the neck was defined as the lowest point of the inferior renal artery. The level with 15% increase in neck diameter compared to the diameter at the level of the lowest renal artery was defined as the end of the neck. The start of the aneurysm sac was defined as the end of the neck, and the end was defined as the first slice cranial to the apex of the aortic bifurcation. The *α*-angle was defined as the angle between the suprarenal aorta and the neck of the aneurysm. The *β*-angle was defined as the angle between the neck and the aneurysm sac.

All measurements were performed by two independent researchers (L.H. and T.L.) and tested for interobserver variability. Outliers and differences were remeasured for accuracy and corrected if necessary.

### 2.4. Statistical Analysis

Data collection and analysis were performed using IBM SPSS Statistics version 21.0 (IBM Corporation, Armonk, NY, USA). All data were checked for outliers and reevaluated for correctness. The Wilcoxon signed rank test was used to test for differences in anatomy both preoperatively and postoperatively as well as during the cardiac cycle. The values *p* < 0.01 were considered statistically significant. All measurement data are presented using medians and interquartile ranges (IQR, Q25–Q75).

Interobserver variability was measured using the intraclass correlation coefficient (ICC). The two‐way random effects model on absolute agreement was used. Using the Landis and Koch interpretation for the kappa value, agreement was scored as poor (<0.20), fair (0.21–0.40), moderate (0.41–0.60), good (0.61–0.80), and perfect (0.81–1.00).

## 3. Results

### 3.1. Patient Data

Patient demographics are presented in Table 1.

The study included 8 patients suitable for elective AAA repair using the Nellix endovascular sealing system (Endologix, Irvine, CA, USA), of which 6 (75%) were male, with a median age of 75.5 (Q25–Q75: 62.2–83.1) years. The maximum AAA diameter was 58.5 mm (range 42.0–75.1 mm). A median volume of 83 mL (range 50–135 mL) of the polymer was used to reach a mean fill pressure of 185 mmHg (range 166–220 mmHg). During implantation, one patient that had a type Ia endoleak caused by underfilling of the endobags had a secondary fill, resolving the issue. In another patient, concomitant angioplasty and stenting of the left superficial femoral artery were performed using a self-expanding nitinol stent (Smart Flex, Cordis Corp., Bridgewater, NJ, USA) because of a flow-limiting stenosis.

The median procedure time was 75 minutes (range 49–101 min), and patients were admitted for a median of 2 days (range 2–4 days). One patient underwent a successful reintervention for a common femoral artery occlusion one day after surgery. Using intra-arterial thrombolysis, the occlusion was dissolved, and the patient was discharged four days after surgery and had a further uneventful follow-up. One patient developed an occlusion of the left popliteal artery seven days after the operation which was successfully treated with thrombolysis. All other patients underwent an uneventful recovery, and no major adverse events were seen at discharge and after 6 weeks of follow-up. The median follow-up was 25 months (range 6–36 months). Within this period no patients died, and there was one patient that presented with a distal migration of ≥5 mm without clear signs of a type Ia endoleak.

### 3.2. Interobserver Variability

The median ICC for continuous variables to assess interobserver variability was 0.68 (range 0.50–0.78) and was therefore classified as good according to Landis and Koch interpretation of ICC.

### 3.3. Neck Anatomy

The changes in neck anatomy are displayed in Table 2.

There were no significant changes observed in the infrarenal neck area during the cardiac cycle in both the pre- and postoperative CT scans. Almost all variables were higher in the diastolic heart phase except for neck length and the lumen volume. With regard to neck angulation, there are some slight changes in both alpha and beta angles and the angulation of the renal arteries during the cardiac cycle, without statistical significance, and with a wide variability in both the pre- and postoperative scans.

When comparing the pre- and postoperative CT scans, a significant increase in neck diameter and surface was observed after EVAS in both the diastolic and systolic phases. In addition, the neck length significantly decreased after EVAS between both phases. The sealing length, defined as the start of the endobag to the start of the aneurysm, was 23.0 mm (range 4.8–34.1 mm) at the peak diastolic phase and 18.6 mm (range 6.3–36.3 mm) at the peak systolic phase (*p*=0.674).

### 3.4. AAA Sac Anatomy

In preoperative CT scans, there were no significant changes during the cardiac cycle in the aneurysm sac (Table 3). In the postoperative scan, we observed a slight but significant change of the luminal volume in the cardiac cycle that was larger in the peak diastolic phase (±6 mL).

When comparing the pre- and postoperative scans, there was a significant increase in the luminal volume after EVAS with a significant decrease in thrombus volume in both the systolic and diastolic phases. The total sac volume was significantly increased in the peak systolic phase. In this phase, the AAA length was also increased in contrast to the diastolic phase.

### 3.5. Iliac Artery Anatomy

During the cardiac cycle, there were no significant changes observed in the anatomy of the iliac arteries in either preoperative or postoperative scans (Table 4), except for the diameter and area of the distal right common iliac artery.

When comparing the pre- and postprocedural imaging, there were also no significant differences except for the diameter of the right common iliac artery, which was larger after implantation when measured in the diastolic phase. The total angulation of both iliac arteries was lower after endovascular sealing as compared to preoperative, albeit nonsignificant.

## 4. Discussion

The current study aimed to investigate the dynamic behavior of aortic anatomy in patients undergoing EVAS. Measurements were performed according to current reporting standards for conventional CT scans and adapted for dynamic CT scanning based on studies performed in a comparable study setting [7, 8]. The current data contribute to the knowledge on this technology, as they suggest that the use of dynamic CT scanning is not indicated in the follow-up of patients that were treated with Nellix in the past and also identified a decrease in neck length after placement as a potential risk factor.

In May 2022, Endologix announced in a targeted communication that the production of Nellix devices and its accessories had stopped (Endologix, Nellix End of Life Communication, 10 May 2022). In a recent meta-analysis including 703 patients from seven studies with a mean follow-up of more than two years (range 24–72 months), the pooled estimated incidence of type 1 endoleak, migration, and re-intervention were 25%, 22%, and 27%, respectively [9]. Although the technology is no longer used in clinical practice, many patients are still under surveillance. Recently, the European Society for Vascular Surgery came out with a focused update on their guidelines, recommending that patients should be enrolled in enhanced follow-up using conventional CTA and duplex ultrasound. A potential role of ECG-gated CTA was not discussed [5].

The data presented in this study suggest that the EVAS reconstruction was stable during the cardiac cycle in these patients, as we found in general no significant changes during the cardiac cycle in both the pre- as well as the postoperative state. These results, however, were measured in the postoperative period and, as a consequence do not ensure the longitudinal stability of the reconstruction. It is known that in patients, particularly when treated outside the IFU, stent migration may occur, leading to a proximal endoleak [9]. Root cause analysis of migration had indicated lateralization of the stents as the driving factor of migration, leading to a refinement in the instructions for use in 2018. The maximum aneurysm sac/maximum flow lumen diameter ratio became a parameter in order to increase the stiffness of the reconstruction [1]. In the current study, the reconstruction lead to a decrease in neck length compared with the CT before surgery. Whether this may have contributed to the risk on migration cannot be concluded from the current data. The current population had a median follow-up of 2-years and within that timeframe one patient presented with a migration of more than 5 mm. In this patient, there was no significant motion during the cardiac cycle. Therefore, this may suggest that an ECG-gated CT scan in the direct postoperative phase is not predictive for the occurrence of migration. To date, no longitudinal studies have been performed using ECG-gated CT scanning, but this would certainly be of value as increased movement during the cardiac cycle might provide an early sign of instability.

The Nellix device itself caused significant changes in the aortic anatomy, particularly with regard to an increased neck diameter, surface, and length, and to the aortic volumes, including the luminal and thrombus volumes. Previous studies which have examined the post-EVAS changes of the anatomy confirm these findings [10]. It has been shown that implantation of the stents leads to changes to the iliac anatomy and the volumes of the aneurysm and thrombus, consistent with the current observations.

All of these changes are likely to be caused by the polymer-filled endobags. The pressurization of these endobags may squeeze the thrombus, declining its volume and increasing the flow volume, and could exert a pressure on the arterial wall, leading to an increase in neck size and AAA volume. These observations emphasize the difference of the EVAS technique when compared to standard EVAR. The implications of these observations, however, are yet unknown. As an unexpected observation, Berg et al. described a lower incidence of the postimplant syndrome after EVAS when compared to EVAR [11]. Whether this is related to the absence of fresh thrombus and/or the decrease in thrombus volume remains to be investigated, but a biological impact of thrombus cannot be excluded.

The current study has limitations. First, the sample size was relatively small. Second, we used only two series of the ECG-gated CT scan. The scans were performed with a total of ten phases during the cardiac cycle. An analysis of all ten phases might have provided more data. However, to attain for the anatomy in its' maximum range, the diastolic and systolic pressures are chosen as the best phases for analysis as they are the phases with both the minimal and maximum changes during the heart cycle. Moreover, CT scans were only available in the early postoperative phase. This would have been relevant, for example, to see if the decrease in neck length after EVAS would be stable in time or would be progressive. The interobserver variability of this study was small, as little to no changes had to be made after secondary measurement of all variables. Software automatization of the CLL is mostly to credit for the accuracy, as measurement can be reproduced in a consistent way.

## 5. Conclusion

This study shows that the cardiac cycle has little to no effect on the morphology of the arteries both before and shortly after EVAS, and therefore there is probably not a role for ECG-gated CTA in enhanced surveillance programs. EVAS itself has a significant impact on anatomy, particularly the neck diameter, length, and volumes of the AAA.

## Figures and Tables

**Table 1:** Patient demographics and intraoperative data.

	*N* (%)
Hypertension	6 (75.0)
Diabetes mellitus	3 (37.5)
Cardiac disease	4 (50.0)
Pulmonary disease	4 (50.0)
Renal insufficiency	1 (12.5)
Cerebral vascular accident	2 (25.0)
Maximum aortic aneurysm diameter (mm)	58.5 (42.0–75.1)
Use of concomitant stents	1 (12.5)
Total polymer volume (mL)	83 (50–135)
Polymer fill pressure (mmHg)	185 (166–220)
Procedure time (min)	75 (49–101)

**Table 2:** Preoperative (PRE) versus postoperative (POST) changes in neck anatomy.

*N* = 8	PRE 20 (%)	PRE 78 (%)	Difference preimplantation	*P*values	POST 20 (%)	POST 78 (%)	Difference postimplantation	*P*values	*P*values PRE vs POST 20 (%)	*P*values PRE vs POST 78 (%)
Suprarenal diameter (mm)	27.8 (26.9–30.9)	29.1 (27.6–31.4)	1.3	0.089	28.8 (27.0–30.1)	28.4 (26.5–30.8)	−0.04	0.618	0.283	0.684
Area suprarenal diameter (mm^2^)	567.0 (474.8–677.1)	604.9 (491.0–696.5)	37.9	0.208	610.4 (540.6–668.3)	589.5 (512.8–663.4)	−20.9	0.305	0.124	0.945
Infrarenal diameter (mm)	22.7 (20.9–27.8)	24.4 (23.5–29.8)	1.7	0.634	27.0 (25.8–34.7)	27.6 (26.4–32.7)	0.6	0.636	<0.001	<0.001
Area of infrarenal diameter (mm^2^)	426.1 (376.6–648.5)	435.0 (405.1–657.7)	8.9	0.409	529.6 (492.9–870.4)	571.5 (469.7–764.9)	41.9	0.469	<0.001	<0.001
Diameter beneath lowest RA (mm)	26.3 (22.0–27.8)	25.1 (24.6–27.3)	−1.2	0.786	26.4 (24.1–30.1)	27.6 (25.8–31.9)	1.2	0.393	0.038	0.044
*α* angle (°)	12.5 (8.0–31.0)	12.5 (7.0–30.8)	0	—	11.5 (7.8–19.3)	19.0 (7.0–30.0)	7.5	0.243	0.140	0.887
*β* angle (°)	14.0 (7.8–18.8)	16.5 (6.5–24.0)	2.5	0.404	9.5 (6–15.5)	6.0 (3.3–15.5)	−3.5	0.522	0.243	0.066
Neck length (mm)	27.0 (13.3–34.1)	25.6 (13.5–34.6)	−1.4	0.840	21.6 (10.0–32.8)	19.5 (8.3–33.8)	−2.1	0.701	0.001	0.044
Neck total volume (mL)	9.4 (6.2–18.1)	9.9 (6.9–15.8)	0.5	0.876	9.3 (4.8–20.6)	9.9 (4.3–17.5)	0.6	0.134	0.434	0.890
Neck lumen volume (mL)	7.4 (4.6–14.7)	6.4 (5.2–12.7)	−1	0.240	7.4 (3.6–14.4)	8.7 (3.5–14.0)	1.3	0.661	0.606	0.662
Right renal angulation (°)	44.0 (39.5–69.0)	49.5 (39.8–72.5)	5.5	0.142	43.5 (38.0–54.8)	50.0 (28.0–58.3)	6.5	0.643	0.838	0.385
Left renal angulation (°)	52.5 (35.8–69.5)	53.5 (36.8–67.8)	1.0	0.866	52.5 (38.8–76.5)	56.0 (40.8–61.0)	3.5	0.675	0.628	0.892

Data are displayed as median, followed by interquartile range (IQR). ^*∗*^Difference in significance in *p* values when preimplantation and postimplantation values were compared.

**Table 3:** Preoperative (PRE) versus postoperative (POST) changes in sack anatomy.

*N* = 8	PRE 20 (%)	PRE 78 (%)	Difference	*P*values	POST 20 (%)	POST 78 (%)	Difference	*P*values	*P*values PRE vs POST 20 (%)	*P*values PRE vs POST 78 (%)
Aneurysm length (mm)	121.2 (82.0–128.3)	116.7 (87.3–131.6)	−4.5	0.272	117.0 (100.3–137.7)	124.6 (96.1–136.8)	7.6	0.454	0.079	0.006
Max angulation aorta (°)	31.0 (25.3–34.0)	27.0 (20.0–34.8)	−4.0	0.434	25.0 (13.8–31.3)	24.5 (12.5–31.8)	−0.5	0.736	0.071	0.196
Max diameter (mm)	65.1 (59.3–66.7)	64.4 (58.9–68.4)	−0.7	0.984	63.2 (60.6–67.7)	62.5 (60.6–69.0)	−0.7	0.724	0.634	0.822
Area on max diameter (mm^2^)	3007.7 (2633.6–3288.0)	3033.4 (2540.3–3448.7)	25.7	0.876	2922.8 (2630.3–3268.2)	2825.2 (2640.5–3399.1)	−97.7	0.759	0.823	0.881
Total volume sac (mL)	238.2 (129.7–270.4)	232.2 (127.4–273.2)	−6.0	0.567	233.2 (150.4–284.1)	236.1 (143.1–278.8)	2.9	0.409	0.062	0.015
Lumen volume sac (mL)	77.0 (68.9–95.2)	75.1 (63.2–91.2)	−2.0	0.146	109.2 (91.3–138.7)	103.4 (87.7–129.0)	−5.8	0.014^*∗*^	<0.001	<0.001
Thrombus volume (mL)	144.8 (64.7–190.3)	151.2 (62.3–192.9)	6.3	0.692	116.2 (60.7–154.0)	121.1 (58.2–160.0)	4.9	0.231	0.009	0.037

Data are displayed as median, followed by interquartile range (IQR). ^*∗*^Difference in significance in *p* values when preimplantation and postimplantation values were compared.

**Table 4:** Preoperative (PRE) versus postoperative (POST) changes in iliac anatomy.

*N* = 8	PRE 20%	PRE 78%	Difference	*P*values	POST 20 (%)	POST 78 (%)	Difference	*P*values	*P*values PRE vs POST 20%	*P*values PRE vs POST 78%
RCIA angle	20.0 (11.5–36.5)	28.0 (9.3–36.3)	8.0	0.690	14.5 (6.3–26.0)	12.0 (7.8–24.0)	−2.5	0.682	0.197	0.180
LCIA angle	23.5 (13.3–39.8)	25.5 (21–34.5)	2.0	0.836	12.5 (8.3–17)	13.5 (11.3–27.5)	1.0	0.833	0.263	0.100
Proximal RCIA diameter (mm)	15.8 (12.3–20.9)	17.1 (13.7–21.0)	1.3	0.095	18.6 (16.6–27.4)	17.6 (16.5–25.7)	−1.1	0.166	0.021	0.104
Area on prox. RCIA diameter (mm^2^)	178.3 (115.1–325.9)	207.8 (132.8–319.1)	29.5	0.232	245.1 (198.4–561.5)	222.6 (206.2–479.3)	−22.6	0.179	0.019	0.235
Proximal LCIA diameter (mm)	17.3 (13.0–27.9)	17.3 (12.8–28.9)	0.1	0.223	18.4 (16.8–22.4)	18.2 (16.2–23.2)	−0.2	0.862	0.646	0.544
Area at prox. LCIA diameter (mm^2^)	210.1 (126.1–576.2)	203.3 (115.5–615.2)	−6.8	0.257	243.6 (195.9–305.9)	245.6 (193.7–407.4)	2.0	0.259	0.438	0.451
Distal diameter RCIA (mm)	19.7 (14.1–24.7)	19.1 (15.0–23.2)	−0.6	0.497	20.6 (16.0–24.4)	19.3 (15.8–23.0)	−1.3	0.022	0.082	0.706
Area on distal RCIA diameter	290.3 (150.1–430.5)	265.1 (146.4–391.1)	−25.2	0.448	326.3 (173.7–421.0)	271.8 (168.9–374.7)	−54.5	0.027	0.213	0.782
Distal diameter LCIA (mm)	18.6 (14.4–26.7)	18.5 (13.8–26.2)	−0.1	0.341	20.3 (17.1–26.6)	20.3 (17.1–26.6)	−1.2	0.381	0.078	0.027
Area on distal LCIA diameter (mm^2^)	248.2 (143.0–483.2)	248.1 (138.3–486.5)	−0.1	0.563	293.6 (134.45–452.8)	272.8 (126.0–419.6)	−20.6	0.908	0.027	0.011

Data are displayed as median, followed by interquartile range (IQR). ^*∗*^Difference in significance in *p* values when preimplantation and postimplantation values were compared.

## Data Availability

The datasets used and/or analyzed during the current study are available from the corresponding author on reasonable request.
